# Preventing Type 2 Diabetes in At-Risk Cardiorenal Populations

**DOI:** 10.7759/cureus.113529

**Published:** 2026-07-28

**Authors:** Diego de Silva, Achille Peiris

**Affiliations:** 1 Nephrology, Balboa Nephrology Medical Group, San Diego, USA

**Keywords:** albuminuria, cardiorenal, cardiovascular-kidney-metabolic syndrome, chronic kidney disease, prediabetes, type 2 diabetes prevention

## Abstract

The convergence of cardiovascular disease, chronic kidney disease, and type 2 diabetes, collectively known as cardiovascular-kidney-metabolic syndrome, affects the majority of US adults. That construct spans a broader population than kidney disease alone, and this review is concerned with a narrower one: patients with chronic kidney disease who are prediabetic or otherwise at risk for incident type 2 diabetes. In these patients, prediabetes represents both a warning and an opportunity. Dysglycemia is common, its progression is detectable early, and preventing it is expected to benefit both the heart and the kidney, a benefit inferred from the mechanisms the two organs share rather than demonstrated directly in outcome trials in this population. Prediabetes is not uniform, and rates of progression and regression vary. Prevention here is not a metabolic problem applied to sicker patients. It is a cardiorenal strategy, because dysglycemia contributes to several of the mechanisms coupling the heart and kidney. This review examines the evidence for diabetes prevention in patients with chronic kidney disease, the identification of patients at highest risk, and the operational structure required to translate evidence into practice. Lifestyle modification remains the foundation and is supported by decades of durable evidence, adapted to the dietary and functional constraints of kidney disease, though real-world adherence falls short of what trial conditions achieved. Metformin is a reasonable pharmacologic addition in selected patients. The agents that have most powerfully transformed cardiorenal medicine, sodium-glucose cotransporter-2 inhibitors and glucagon-like peptide-1 receptor agonists, are not diabetes prevention agents on current evidence, but the prediabetic patient often already qualifies for them on renal and cardiac grounds alone. This fact is frequently missed in clinical practice. Prevention succeeds when the modifiable drivers of disease are addressed early and without deferral, when organ-protective therapy is initiated on the indications that exist rather than withheld pending a diagnosis not yet made, and when responsibility is shared explicitly between nephrology and cardiology rather than defaulted at the interface between them. The principal barriers to prevention are organizational rather than clinical.

## Introduction and background

Few conditions in chronic disease are simultaneously as prevalent, as costly, and as amenable to prevention as the convergence of metabolic and cardiorenal disease. Clinicians have long observed that dysglycemia, kidney disease, and cardiovascular disease travel together, yet for most of the modern era, they have been managed as separate diagnoses belonging to separate specialties. The American Heart Association formalized the alternative in 2023, defining cardiovascular-kidney-metabolic (CKM) syndrome as the staged overlap of dysfunctional adiposity, metabolic risk factors, chronic kidney disease (CKD), and cardiovascular disease, framing these not as coincident comorbidities but as expressions of a shared pathophysiologic continuum [1,2]. The staging system itself is a clinical and organizational framework rather than a mechanistic model; the biological mechanisms linking these organ systems are established separately in the physiological literature, and the staging is the clinical structure built around them. The multisociety guideline that followed in 2026 built preventive practice around that framework [3]. Where it places dysglycemia is what makes it consequential here. Prediabetes appears at the earliest symptomatic stage of the syndrome, alongside excess or dysfunctional adipose tissue, well before metabolic risk factors are established, before kidney disease is evident, and long before any cardiovascular event [3]. The major cardiology, nephrology, and diabetes societies have therefore jointly located the earliest actionable signal of cardiorenal disease within the metabolic domain. CKM staging encompasses a far broader population than kidney disease alone, extending to patients with metabolic risk factors and no renal involvement. This review narrows from that construct to patients with established CKD, because it is in this group that the cardiovascular stakes of incident dysglycemia are highest and the case for a distinct prevention strategy is strongest.

The epidemiology justifies the attention. Between 90% and 95% of US adults fall within CKM stages 1 through 4, which is to say that the large majority already carry at least one component of the syndrome [3]. That figure reflects the breadth of the staging framework, which begins with excess or dysfunctional adiposity, and should not be read as indicating that most adults have advanced cardiorenal disease. Within that population, the overlap between metabolic and renal disease is substantial. Type 2 diabetes (T2D) is the leading cause of CKD, and national survey data estimate the prevalence of CKD among US adults with T2D at approximately 43%, rising above 60% among those aged 65 and older [4]. These are not two populations that intersect incidentally. They are largely a single population observed through two diagnostic lenses, and the clinician treating one is, in most cases, already treating the other. The prevalence figures cited here are drawn from US cohorts and US guideline frameworks. The pathophysiology and the prevention principles that follow are not US-specific, and the Kidney Disease: Improving Global Outcomes (KDIGO) staging on which much of the later discussion rests is international in scope.

For the patient who carries both diagnoses, the predominant threat is cardiovascular. Heart failure and atherosclerotic cardiovascular disease are the leading causes of death once diabetes and kidney disease coexist [2]. CKD is among the most powerful independent multipliers of cardiovascular risk in clinical medicine, and dysglycemia compounds it further. A review of diabetes prevention in this setting is therefore as relevant to the cardiologist as to the nephrologist, and the emphasis throughout this paper is weighted accordingly toward cardiovascular outcomes. The kidney is the accelerant of risk. The cardiovascular system is where that risk is realized.

What makes the burden tractable is that its earliest stage is also its most modifiable. Prediabetes is not diabetes. A reduced estimated glomerular filtration rate (eGFR) is not kidney failure. Albuminuria is not yet a cardiovascular event. Each is a point at which the trajectory can still be altered, and each is detectable with inexpensive tests that are, in most cases, already being ordered [5]. Prediabetes is not a uniform state, and its natural history varies: some patients progress to diabetes, others regress to normoglycemia, and baseline characteristics influence which path a given patient follows [3]. Targeting therefore matters as much as detection. The obstacle is less often the absence of evidence than the absence of an owner, because the cardiorenal patient sits between two specialties and the dysglycemia threatening both organs belongs unambiguously to neither. This review addresses, in turn, the shared mechanisms coupling the heart and kidney in dysglycemia, the prevention strategies supported by evidence and the strength of that evidence, the identification of patients in whom prevention carries the greatest return, and the coordination required to deliver it. This review is narrative in approach, drawing on current guidelines from KDIGO, the American Diabetes Association, and the American Heart Association together with the pivotal randomized trials and meta-analyses in the field, selected for topical relevance rather than through a predefined systematic search. The thesis is simple. In the cardiorenal patient, preventing T2D is among the highest-yield interventions available to the clinician, and it remains within reach.

## Review

Pathophysiology: the cardiorenal link

The Shared Substrate

In practice, heart and kidney disease tend to reinforce each other. They share a common vascular bed, a common neurohormonal regulatory system, and a common susceptibility to metabolic injury. The CKM construct organizes this clinically, holding that their co-occurrence is not an epidemiologic coincidence to be managed as separate diagnoses [1,2]. The mechanisms that account for it are described below and rest on the physiological literature rather than on the staging framework itself. When a patient with CKD develops T2D, the new diagnosis does not merely coexist with the established one. It initiates a cascade that injures both organs concurrently, and this is the mechanistic basis for prevention in this population.

The relationship is bidirectional, and the direction less often discussed is the one that matters most for targeting. Reduced kidney function actively promotes dysglycemia. Impaired renal clearance of insulin alters its kinetics. Uremic solutes interfere with insulin signaling. Chronic low-grade inflammation and metabolic acidosis each impair insulin sensitivity. The muscle wasting and reduced activity that accompany advancing CKD diminish the skeletal muscle mass responsible for most insulin-mediated glucose disposal. The patient with kidney disease therefore arrives at the threshold of dysglycemia already primed to cross it. Prevention here is not the application of general population strategy to a sicker cohort. It is intervention in a patient whose physiology is working against the clinician.

Cardiovascular Mechanisms

In this population, the cardiovascular consequences predominate, and several mechanisms converge on the myocardium and vasculature. Insulin resistance and hyperglycemia promote endothelial dysfunction, oxidative stress, and a prothrombotic, proinflammatory state. Within the uremic milieu, these processes are superimposed on disordered mineral metabolism and vascular calcification, producing atherosclerosis that is more diffuse, more heavily calcified, and less amenable to revascularization than in patients with normal renal function [2]. Pressure and volume overload, anemia, and the mineral and bone disturbances of CKD drive left ventricular hypertrophy and diastolic dysfunction, while diabetes contributes a distinct cardiomyopathy marked by interstitial fibrosis and impaired myocardial energetics. The resulting ventricle is predisposed to heart failure and particularly to the preserved-ejection-fraction phenotype that dominates this population and for which therapeutic options remain most limited.

Beneath these processes lies chronic neurohormonal activation. The renin-angiotensin-aldosterone system and the sympathetic nervous system are persistently activated in both CKD and diabetes, raising afterload, promoting sodium retention, and driving fibrosis in the myocardium and kidney in parallel [2]. Mineralocorticoid receptor overactivation contributes independently to inflammation and fibrosis in both organs, which is the mechanistic basis for the benefit observed with nonsteroidal mineralocorticoid receptor antagonism [6]. The clinical translation is direct. A patient with reduced eGFR, albuminuria, and dysglycemia carries an exceptionally high risk of major adverse cardiovascular events, and that risk rises steeply and continuously as renal function declines [5].

Renal Mechanisms and the Albuminuria Signal

Dysglycemia injures the kidney through mechanisms that are well characterized and, importantly, therapeutically addressable. Hyperglycemia produces glomerular hyperfiltration by altering tubuloglomerular feedback: increased proximal reabsorption of sodium and glucose reduces distal sodium delivery to the macula densa, prompting afferent arteriolar vasodilation and raising intraglomerular pressure. Sustained, this hemodynamic stress injures the podocyte, whose loss is largely irreversible. Mesangial expansion and the deposition of advanced glycation end-products drive fibrosis in parallel. Albuminuria rises, and the slope of eGFR decline steepens, progressing faster than either condition would produce alone. This mechanism is worth stating precisely, because it is the reason a major class of contemporary therapy works. Sodium-glucose cotransporter-2 (SGLT2) inhibition restores distal sodium delivery, reinstates tubuloglomerular feedback, and lowers intraglomerular pressure, which explains both the expected early dip in eGFR on initiation and the durable protection that follows [7,8].

Albuminuria warrants separate emphasis, because it is the single parameter that speaks to both organs at once. It arises from glomerular and tubular pathophysiology, including a breach of the filtration barrier and altered tubular handling of filtered protein, and it also reflects generalized endothelial dysfunction, of which the glomerulus is one compartment where the defect becomes measurable. This is why albuminuria predicts cardiovascular events and not merely renal ones and why it does so at any level of kidney function, including in patients whose eGFR remains preserved [5]. A rising albumin-to-creatinine ratio in a patient with normal creatinine is not a nephrologic finding awaiting confirmation. It is an early warning that the vascular endothelium is failing and the kidney is where it showed first.

Implications for Prevention

Assembled, these mechanisms describe a cycle rather than a sequence. Insulin resistance and hyperglycemia injure the glomerulus and the endothelium. Declining renal function worsens insulin resistance, activates the neurohormonal axes, and accelerates vascular and myocardial injury, which in turn compromises renal perfusion. Each turn of the cycle narrows the options available at the next.

Once diabetes is established in a cardiorenal patient, the therapeutic window narrows and management shifts toward slowing a process already well advanced. Prevention operates upstream, before the cycle is fully engaged. Because the injury proceeds through mechanisms common to both organs, an intervention that prevents or delays diabetes would not be expected to act on only one of them. This inference follows from the shared mechanisms described above rather than from trials measuring cardiorenal endpoints after diabetes prevention in this population. This is the principle on which the remainder of the review rests, and it is what establishes diabetes prevention in this population as a cardiorenal strategy rather than a metabolic afterthought.

Prevention strategies

The prevention of T2D in the cardiorenal patient is not a single decision but a graded sequence of them, and the evidence underlying each tier differs enough that candor about those differences distinguishes a credible review from an aspirational one. The sequence runs from behavioral intervention, to pharmacologic prevention where risk is high, to a class of agents that modify cardiorenal risk once dysglycemia is established but that do not, on the available evidence, prevent it.

Lifestyle Modification

Lifestyle modification is the foundation, and its supporting evidence is the most durable in the field. In the Diabetes Prevention Program, a randomized trial of 3,234 adults with prediabetes, an intensive lifestyle intervention reduced the three-year incidence of T2D by 58% (95% CI: 48-66) relative to placebo [9]. The intervention deserves specification, because the word "lifestyle" invites vagueness where the trial was precise. Participants pursued a target of at least 7% body weight loss and a minimum of 150 minutes of moderate physical activity per week, supported by structured, individually delivered behavioral counseling. This was not general advice to eat better and move more. It was a defined program with defined targets, and the effect it produced has not been surpassed by any pharmacologic agent tested for the same purpose.

The persistence of that effect is what makes it relevant to a high-risk population. In long-term follow-up, a protective effect of approximately 24% remained at 21 years, despite relaxation of the intervention itself [10]. A bounded behavioral investment conferred benefit across nearly two decades, a return few interventions in chronic disease can claim. These results were achieved under trial conditions, with structured, individually delivered coaching that is rarely available in routine care. Real-world adherence falls short of what the trial achieved, and the gap is a structural feature of how prevention is delivered rather than a limitation of the evidence itself.

The mechanistic rationale for amplified benefit in the cardiorenal patient is straightforward. Weight loss and physical activity improve peripheral insulin sensitivity, lower blood pressure, improve lipid profiles, and reduce systemic inflammation, addressing several nodes of the shared pathophysiology at once. Weight loss additionally attenuates the glomerular hyperfiltration that accelerates renal decline. The same intervention that lowers the probability of diabetes therefore lowers atherosclerotic and heart failure risk in a group already carrying an excess of both, so the cardiovascular dividend exceeds what would be realized in a metabolically healthy patient.

Two qualifications specific to this population deserve statement, and their routine absence from general reviews of diabetes prevention is precisely why they belong here. The first is dietary. Patients with advancing CKD often operate under restrictions on protein, potassium, phosphate, and sodium, and these collide directly with the dietary patterns most often recommended for diabetes prevention, which tend to be rich in fruit, vegetables, legumes, and whole grains. A clinician who prescribes a diabetes prevention diet without accounting for renal constraints will produce a plan the patient cannot follow, and the resulting nonadherence will be misread as a failure of motivation. Coordination with a renal dietitian is not a refinement here. It is the difference between a deliverable intervention and an abstract one.

The second concerns physical activity. Sarcopenia, uremic fatigue, anemia, and reduced exercise capacity are common in advancing kidney disease, and an activity prescription calibrated to the general population may simply be unattainable. The correct response is adjustment rather than abandonment, since relative benefit is preserved even where the absolute activity level achieved is modest. But the prescription has to be realistic if it is to be followed at all.

Pharmacologic Prevention: Metformin

Where behavioral change proves insufficient or impractical, metformin carries the strongest pharmacologic evidence for prevention. In the same trial, it reduced three-year incidence by 31% (95% CI: 17-43), with a durable effect of approximately 17% at 21 years [9,10]. It acts principally by reducing hepatic gluconeogenesis and improving peripheral insulin sensitivity. It is inexpensive, well tolerated at preserved or moderately reduced kidney function, and cardiovascularly safe.

The effect is not uniform, and the heterogeneity is clinically actionable. Long-term follow-up of the trial population demonstrated that treatment effects varied by baseline characteristics, with the absolute benefit of metformin greater in younger participants and the absolute benefit of lifestyle intervention greater in those with higher baseline fasting glucose and glycated hemoglobin [10]. Metformin is therefore not a uniform second-line default but an agent with an identifiable population in which it approaches the performance of lifestyle intervention.

Two further qualifications are warranted. Metformin is not labeled by the US Food and Drug Administration for diabetes prevention, although it is used for this purpose in practice and supported by professional guidance in selected high-risk patients [11]. The distinction is not pedantic, since it shapes how the intervention is discussed with the patient and documented in the record. More centrally for this population, the principal constraint on metformin is renal. It requires dose reduction as kidney function declines and is generally avoided below an eGFR of 30 mL/min/1.73 m^2^. The historical concern regarding lactic acidosis has been substantially moderated by subsequent evidence, and the agent is now used at levels of renal function once considered prohibitive, but the constraint remains real at the lower end. Long-term use also warrants periodic assessment for vitamin B12 deficiency.

The Cardiorenal Pharmacotherapy Landscape

A complete review must address the agents that have transformed cardiorenal medicine over the past decade and must be precise about what they do and do not prevent. SGLT2 inhibitors and glucagon-like peptide-1 (GLP-1) receptor agonists are not, in the strict sense, diabetes prevention agents. Their pivotal trials enrolled patients with pre-existing T2D, CKD, or heart failure. They matter here for two reasons: the cardiorenal patient who does progress to diabetes now enters a substantially more protective treatment landscape than existed a decade ago, and the magnitude of that protection is itself an argument for earlier identification.

The cardiovascular evidence is the most prominent. In a meta-analysis of SGLT2 inhibitor trials in patients with CKD, treatment reduced cardiovascular death (relative risk: 0.87; 95% CI: 0.79-0.95) and substantially reduced heart failure events (relative risk: 0.67; 95% CI: 0.61-0.75) [7]. The trial-level data are concordant but not identical, and the differences are instructive. In EMPA-KIDNEY, empagliflozin reduced a composite of kidney disease progression and cardiovascular death by 28% (hazard ratio: 0.72; 95% CI: 0.64-0.82), although the benefit was driven by the renal component, and the isolated effect on heart failure hospitalization or cardiovascular death did not reach statistical significance [8]. In CREDENCE, canagliflozin reduced major adverse cardiovascular events (hazard ratio: 0.80; 95% CI: 0.67-0.95) [12]. In DAPA-CKD, dapagliflozin reduced a composite of cardiovascular death and heart failure hospitalization (hazard ratio: 0.71; 95% CI: 0.55-0.92) [13]. The variation tracks differences in enrolled populations and composite endpoints, which argues for reading the class effect from the aggregate rather than from any single trial.

The mechanism of cardiovascular benefit bears directly on the argument of this review. The protection conferred by SGLT2 inhibition is not principally attributable to glucose lowering. Benefit emerges early, before meaningful differences in glycemic control accrue, and it is observed in patients without diabetes. The operative mechanisms appear to be hemodynamic and metabolic rather than glycemic: natriuresis and plasma volume reduction, lowered intraglomerular pressure through restored tubuloglomerular feedback, reduced cardiac preload and afterload, and a shift in myocardial substrate utilization toward more efficient energetics. These agents act on the shared cardiorenal substrate described in "Pathophysiology: the cardiorenal link", which is precisely why their benefit spans both organs.

GLP-1 receptor agonists confer comparable cardiovascular protection. In a network meta-analysis, both classes reduce major adverse cardiovascular events to a similar degree, with a hazard ratio of 0.86 for GLP-1 receptor agonists and 0.89 for SGLT2 inhibitors, while SGLT2 inhibitors show a particular advantage for heart failure hospitalization [14]. The renal contribution is substantive as well: the same agents slow CKD progression, with SGLT2 inhibitors reducing the risk of progression by approximately one quarter in established disease [7]. A single drug class protecting both organs is exactly the organ-spanning effect the cardiorenal framework predicts.

The Limits of the Evidence

Intellectual honesty requires naming what the evidence does not establish, and the gap here is central rather than peripheral.

The trials that define diabetes prevention were conducted in populations selected for dysglycemia, not for CKD. Participants were not enrolled on the basis of reduced eGFR or albuminuria, and the trials were not powered to detect differential effects in a cardiorenal subgroup. Applying these results to the patient with established kidney disease is therefore an extrapolation. It is a well-reasoned one, grounded in shared mechanism and unopposed by any signal that these interventions perform worse under renal impairment, but an extrapolation nonetheless.

The converse gap is equally notable. The agents with the strongest cardiorenal protection in established disease have not been tested for the prevention of incident diabetes in a cardiorenal population. Whether SGLT2 inhibition or GLP-1 receptor agonism would prevent progression from prediabetes to diabetes in a patient with CKD, and whether doing so would translate into fewer cardiovascular and renal events, is unknown. This is the most consequential open question in the field, and it defines a trial that has not yet been done. No dedicated prevention trial in this population has been reported, and the question therefore remains open rather than one awaiting the readout of a study already underway.

The Prevention Hierarchy

The hierarchy is evident once the evidence is set out explicitly. Lifestyle modification is first-line, best supported, and durable across decades, requiring adaptation to the dietary and functional constraints of kidney disease. Metformin is a reasonable pharmacologic addition in an identifiable subgroup, constrained by renal function. The SGLT2 inhibitors and GLP-1 receptor agonists are not prevention agents on current evidence, but they define the protective landscape that follows progression and reinforce the case for early case identification. Identifying those patients is the subject of the next section.

Risk stratification and screening

A prevention strategy is only as effective as the targeting that directs it. An approach validated in a trial cohort fails in practice if clinicians cannot reliably identify the patients positioned to benefit. For the cardiorenal patient, this requires the simultaneous assessment of two signals: the metabolic signal that indicates approaching diabetes and the cardiorenal signal that determines the magnitude of what is at stake.

Assessment of Metabolic Risk

The point of entry is the recognition of prediabetes, the intermediate state preceding diabetes that itself constitutes a window for intervention. Screening rests on three measures, HbA1c, fasting plasma glucose, and the oral glucose tolerance test, with established thresholds defining the prediabetic range [15]. In most patients, HbA1c serves as the convenient default.

The cardiorenal patient imposes a qualification that any rigorous treatment of this subject must state. HbA1c loses reliability in advanced kidney disease, where altered erythrocyte turnover, anemia, and the agents used in its treatment distort the result [15]. In patients with substantially reduced renal function, reliance on HbA1c alone is inadvisable; fasting glucose or oral glucose tolerance testing frequently reflects the true glycemic state more accurately. The point is immediately recognizable to a nephrology or cardiology readership, and its inclusion signals attention to the specific population under discussion.

Assessment of Cardiorenal Risk

Identifying dysglycemia constitutes only half of the assessment. The remainder is the quantification of existing cardiovascular and renal risk, which establishes the urgency of intervention. Here, the framework is well established. Risk in CKD is defined along two axes assessed together, the eGFR and albuminuria, the latter typically expressed as the urine albumin-to-creatinine ratio [5]. Neither measure is sufficient in isolation; their combination is what stratifies risk, and current guidance recommends the assessment of both at least annually in at-risk patients [5,16].

Albuminuria warrants particular emphasis in a review weighted toward cardiovascular outcomes, as it is the clearest instance of a single parameter informative for both organs. At any level of kidney function, the degree of albuminuria independently predicts cardiovascular disease, progression of kidney disease, and mortality [5]. A rising albumin-to-creatinine ratio is not merely a nephrologic finding to be referred onward; it is a cardiovascular signal that warrants the attention of both specialties. This represents the cardiorenal relationship rendered in concrete clinical terms.

Integrated Risk Stratification

The highest-priority patient occupies the intersection: dysglycemic or frankly prediabetic, with reduced eGFR, elevated albuminuria, or both. In this patient, the shared mechanisms of injury are already active, and the cost of progression to diabetes is at its greatest. Contemporary guidance increasingly favors externally validated risk equations incorporating eGFR and albuminuria to estimate an individual's future cardiovascular and kidney failure risk, advancing beyond population-level staging toward individualized stratification [5].

The screening cadence follows from this logic. At-risk patients warrant the assessment of glycemic status, eGFR, and albuminuria at least annually, with shorter intervals where values are changing or where a treatment decision depends on the result [5]. The objective is to identify the patient while the preventive window remains open, before dysglycemia progresses to diabetes and the cardiorenal trajectory steepens. The means of acting on these findings, and of coordinating that action across specialties, is the subject of the following section.

Clinical implementation

A prevention strategy that exists only in principle achieves nothing. The cardiorenal patient is, almost by definition, situated between two specialties, and this interface is precisely where preventive opportunities are lost. The nephrologist monitors renal parameters. The cardiologist manages cardiovascular risk. The dysglycemia that threatens both organs may go unaddressed because neither service fully assumes responsibility for it. Implementation is therefore a question of what gets done, by whom, and in response to which signal.

The Organizing Principle

The operational goal in this population follows directly from the pathophysiology. The injury described in "Pathophysiology: the cardiorenal link" is a self-reinforcing cycle, and the modifiable inputs to that cycle are few and well characterized: blood pressure, dysglycemia, and excess adiposity. The strategy that follows is to attack those inputs early and hard, so that the cycle has no fuel and progression has nowhere to go. This is not a novel principle, but it is unevenly applied, and its underapplication in the cardiorenal patient is the practical failure this section addresses. The therapeutic recommendations that follow are drawn from current guidelines and cited accordingly. The observations on workflow, ownership, and process design are offered as practical judgment rather than as evidence-based recommendations and are identified as such where they appear.

Blood Pressure Control

Blood pressure control is the intervention most likely to be assumed to be handled and least likely to be optimized. Chronic activation of the renin-angiotensin-aldosterone system drives hypertension, cardiac remodeling, and renal fibrosis in parallel, which is why renin-angiotensin system inhibition with an angiotensin-converting enzyme inhibitor or angiotensin receptor blocker remains foundational in the patient with albuminuria [5,16]. Guideline-directed blood pressure control in this population is not simply cardiovascular hygiene. It slows the renal trajectory, and it does so through the same mechanistic axis that drives the cardiac injury.

One point deserves explicit statement because it is a recurring source of inappropriate discontinuation. Current guidance supports continuing an angiotensin-converting enzyme inhibitor or angiotensin receptor blocker even as eGFR falls below 30 mL/min/1.73 m^2^ in patients with albuminuria [5,16]. Withdrawal of renin-angiotensin system inhibition at that threshold, out of concern for the kidney, forfeits both renal and cardiovascular protection at exactly the point where the patient can least afford it.

Glycemic Management

For the patient who has not yet progressed, the prevention hierarchy established in "Prevention strategies" applies directly: structured lifestyle intervention adapted to renal dietary and functional constraints, with metformin as a reasonable pharmacologic addition in the subgroup where it performs best and where renal function permits it. Neither requires specialist supervision to begin, and deferring either pending referral introduces delay the patient cannot afford.

The Overlooked Eligibility

Here lies the most consequential implementation gap in this population, and it is one of missed indications rather than missing evidence.

The patient this review has been describing, with reduced eGFR, elevated albuminuria, and dysglycemia short of diabetes, is frequently already eligible for organ-protective therapy on renal and cardiac grounds alone. Current kidney guidance recommends an SGLT2 inhibitor in CKD with an albumin-to-creatinine ratio at or above 200 mg/g, and in patients with heart failure irrespective of albuminuria, with treatment also suggested at an eGFR between 20 and 45 mL/min/1.73 m^2^ at lower levels of albuminuria [5]. None of these indications requires that the patient have diabetes.

The implication is worth stating plainly, because it is easily missed. These agents are not diabetes prevention therapy, and this review has been careful not to present them as such. But the prediabetic patient with CKD is often a patient who should already be receiving SGLT2 inhibition for cardiorenal protection under existing indications and who is not receiving it. The prescribing decision is anchored to the diabetes diagnosis the patient has not yet made. The clinician waiting for the HbA1c to cross a threshold before initiating an organ-protective agent has misread the indication. The kidney and the heart do not require the diagnosis. They require the eGFR and the albuminuria, which are already there.

In our observation, this eligibility gap operates at scale through three mechanisms, described here as practical explanation rather than as findings from studies of prescribing behavior. First, the electronic health record typically sorts medications by diagnosis, not by indication. A provider searching for kidney-protective therapy finds it under CKD if it is there at all, and many systems require the diabetes diagnosis to surface the agent at all. Second, referral pathways in fragmented care move patients to endocrinology only after the diabetes diagnosis is made, which means the prediabetic patient never reaches the prescriber with the strongest familiarity with these agents. Third, the language in which these medications are discussed in practice centers them on glycemic control. A provider who thinks of SGLT2 inhibitors as diabetes drugs misses that the patient with prediabetes and albuminuria already qualifies.

For the patient who has progressed to T2D, the case is stronger still. An SGLT2 inhibitor is recommended in T2D with CKD down to an eGFR of 20 mL/min/1.73 m^2^, with continuation below that threshold in appropriate patients [5,16]. A GLP-1 receptor agonist with demonstrated cardiovascular benefit is indicated where additional cardiovascular risk reduction, glycemic control, or weight reduction is required [16]. For patients who remain at high residual risk despite maximally tolerated renin-angiotensin system inhibition, a nonsteroidal mineralocorticoid receptor antagonist reduces cardiovascular events and slows renal decline in appropriate candidates and is recommended in T2D with an eGFR above 25 mL/min/1.73 m^2^, normal serum potassium, and persistent albuminuria [5,6].

The unifying principle, consistent with the cardiovascular emphasis of this review, is that the agents that protect the kidney in this population also reduce major adverse cardiovascular events and heart failure hospitalization. Selection and sequencing depend on renal function. The cardiovascular benefit is constant.

Monitoring

Implementation requires a monitoring discipline both services regard as reliable, and one consideration merits explicit statement for the prescribing clinician. A modest early decline in eGFR following the initiation of a renin-angiotensin system inhibitor or an SGLT2 inhibitor is expected and hemodynamic in origin, reflecting precisely the reduction in intraglomerular pressure that produces the long-term benefit. It is not evidence of harm. A larger acute decline, conventionally taken as greater than approximately 30%, exceeds what initiation alone should produce and warrants evaluation rather than reflexive discontinuation [5]. Stating this in advance prevents the unnecessary withdrawal of organ-protective therapy that would otherwise undermine the entire strategy, and such withdrawal is common enough to be worth naming.

Multidisciplinary Coordination

None of the above succeeds without a resolution of responsibility. An operationally efficient division assigns the nephrologist ownership of renal monitoring and kidney-protective therapy and the cardiologist ownership of cardiovascular risk assessment and heart failure management. The dysglycemia and the albuminuria signal belong to both. Treating these as shared, with an explicit understanding that either specialist may initiate lifestyle intervention, prediabetes screening, and first-line prevention, prevents the patient from being lost at the interface between clinics. The coordination model matters more than the identity of the clinician who acts.

Structural Enablers

The obstacles are familiar and predominantly structural: fragmented care, referral delay, inconsistent testing, and diffuse ownership of prevention. None is resolved by clinical evidence alone. Each is resolved through deliberate process design.

The practical instruments are unglamorous, and in our judgment effective, though we are not aware of trial evidence evaluating them in this population. Electronic record standing orders ensure that eGFR and albuminuria are measured together, addressing the asymmetry whereby creatinine is always available and urine albumin-to-creatinine ratio frequently is not. This automated pairing makes the two-axis risk stratification described in "Risk stratification and screening" routine rather than aspirational. Decision support flags a rising albumin-to-creatinine ratio or a prediabetic HbA1c to both services simultaneously, rather than to a single inbox that may or may not route it correctly. Shared care pathways specify in advance which service initiates which therapy, and standing agreements permit first-line prevention to begin without awaiting formal cross-referral. A template in the electronic health record documenting an expected early eGFR dip on initiation of therapy prevents its misinterpretation as treatment failure and subsequent drug discontinuation. These tools are straightforward. Their implementation requires commitment, because they demand coordination meetings and workflow redesign that add to administrative work in the short term.

A practice designing its care model deliberately holds a real advantage here over one retrofitting solutions onto inherited fragmentation. The friction is front-loaded, but it is finite. The payoff we would expect, measured in fewer progression events and fewer emergency department visits for hyperglycemic crisis or decompensated heart failure, follows from the therapeutic evidence cited above rather than from the direct evaluation of these workflow measures.

The argument is not that a single clinic must perform every function. It is that the preventable patient must not be the one for whom no one assumes responsibility. With ownership defined, with the modifiable drivers attacked early, with therapy matched to renal function, and with monitoring protocols agreed upon in advance, prevention in the cardiorenal patient ceases to be aspirational and becomes routine.

## Conclusions

The argument of this review reduces to a single principle. In the cardiorenal patient, the prevention of T2D is not a metabolic afterthought but a cardiovascular and renal intervention in its own right. Because the heart and kidney are injured through several shared mechanisms, intervention upstream would be expected to benefit both, an inference that rests on those mechanisms rather than on trials measuring cardiorenal endpoints after diabetes prevention in this population. That expectation is why the return on prevention here is plausibly greater than in nearly any other group and why the cost of inaction is counted in clinical events rather than laboratory values.

Three conclusions carry the greatest practical weight.

The prevention hierarchy is clear, and it is evidence-based. Lifestyle modification is first-line, best supported, and durable across decades, provided it is adapted to the dietary and functional realities of kidney disease rather than prescribed as though those realities did not exist. Metformin is a reasonable addition in an identifiable subgroup, constrained principally by renal function. The agents that have transformed cardiorenal medicine are not, on current evidence, prevention agents, and this review has declined to present them as such.

Targeting is decisive. The patient who matters most is identified by reading glycemic status alongside eGFR and albuminuria, and albuminuria is the parameter that speaks to both organs at once, reflecting glomerular and tubular injury together with endothelial dysfunction and predicting cardiovascular as well as renal events at any level of kidney function. The patient with three separately unremarkable values may be the patient at greatest risk, and the failure to read those values together is the most common way that risk is missed.

Prevention here is as much an operational problem as a clinical one. It succeeds when the modifiable drivers, blood pressure and dysglycemia, are attacked early and without deferral; when organ-protective therapy is initiated on the indications that actually exist rather than withheld pending a diagnosis the patient has not yet made; when an expected hemodynamic dip in eGFR is not mistaken for harm; and when nephrology and cardiology agree in advance on who acts, so that the patient at the interface between them is not the patient no one owns.

The direction of the field is encouraging. The therapeutic landscape has shifted decisively toward agents that protect the heart and kidney in concert, which validates the cardiorenal framework at the level of therapy and not merely of theory. The open questions are correspondingly sharper. Whether these agents prevent the onset of diabetes in the cardiorenal patient, rather than modifying its consequences once established, remains untested, and it is the trial the field most needs. How prevention is integrated into routine cardiorenal care, rather than maintained as a separate and easily deferred workflow, remains largely an implementation problem awaiting evidence.

Prevention has long been the least conspicuous domain of medicine and frequently the most consequential. In the cardiorenal patient, it is also among the highest-yield, and delivering it requires no discovery. It requires organization.
